# FLAIR-hyperintense lesions in anti-MOG-associated encephalitis with seizures overlaying anti-N-methyl-D-aspartate receptor encephalitis: a case report and literature review

**DOI:** 10.3389/fimmu.2023.1149987

**Published:** 2023-04-17

**Authors:** Jia-Xin Yang, Miao-Miao Yang, Yu-Juan Han, Cai-Hong Gao, Jie Cao

**Affiliations:** Neurology Department, Neuromedical Center, First Hospital of Jilin University, Changchun, China

**Keywords:** MOG ab-positive CCE, anti-N-methyl-D-aspartate receptor encephalitis, symptoms, demyelinating disease, FLAIR

## Abstract

**Background:**

FLAIR-hyperintense lesions in anti-MOG-associated encephalitis with seizures (FLAMES) has been identified increasingly frequently in recent years. However, this rare MOG antibody disease may coexist with anti-N-methyl-D-aspartate receptor encephalitis (anti-NMDARe), in an overlap syndrome with unknown clinical features and prognosis.

**Methods:**

We report a new case of this overlap syndrome and present a systematic review of similar cases in the literature to provide information on the clinical presentation, MRI features, EGG abnormalities, treatment, and prognosis of patients with this rare syndrome.

**Results:**

A total of 12 patients were analyzed in the study. The most common clinical manifestations of FLAMES overlaid with anti-NMDARe were epilepsy (12/12), headache (11/12), and fever (10/12). Increases in intracranial pressure (median: 262.5 mmH_2_O, range: 150–380 mmH_2_O), cerebrospinal fluid (CSF) leukocyte count (median: 128×10^6^/L, range: 1-610×10^6^/L), and protein level (median: 0.48 g/L) were also observed. The median CSF anti-NMDAR antibody titer was 1:10 (1:1–1:32), while the median serum MOG antibody titer was 1:32 (1:10–1:1024). Seven cases exhibited unilateral cortical FLAIR hyperintensity, and five cases (42%) had bilateral cortical FLAIR hyperintensity, including four cases involving the bilateral medial frontal lobes. Of the 12 patients, five showed lesions at other sites (e.g., the brainstem, corpus callosum, or frontal orbital gyrus) before or after the development of cortical encephalitis. EEG showed slow waves in four cases, spike–slow waves in two cases, an epileptiform pattern in one case, and normal waves in two cases. The median number of relapses was two. Over a mean follow-up period of 18.5 months, only one patient experienced residual visual impairment, while the remaining 11 patients had good prognoses.

**Conclusion:**

FLAMES alone is difficult to distinguish from overlap syndrome based on clinical features. However, FLAMES with bilateral medial frontal lobe involvement suggests the presence of the overlap syndrome.

## Introduction

Myelin oligodendrocyte glycoprotein (MOG) is a protein expressed in the outermost layer of the myelin sheath of the central nervous system. MOG antibody-associated disease (MOGAD) is gradually becoming recognized as a new independent spectrum of disease. The most common clinical manifestations of MOGAD are optic neuritis (ON), myelitis, and acute disseminated encephalomyelitis, while cortical encephalitis, demyelination of the brainstem and cerebellum, and progressive white matter damage are rare (1). The term “FLAIR-hyperintense lesions in anti-MOG-associated encephalitis with seizures” (FLAMES) was introduced by Budhram in 2019. Although this was initially reported as unilateral cortical encephalitis, the presence of bilateral cortical involvement and meningeal inflammation in a subset of cases suggests a broader disease spectrum (2). Atypical manifestations of demyelination, such as fever, headache, epilepsy, and aphasia, are common in FLAMES.

Anti-NMDAR encephalitis is one of the most common types of autoimmune encephalitis and is associated with the presence of NMDAR subunit 1 (NR1) antibodies (3). Patients may experience abnormal mental behavior, involuntary movements of the mouth, and central hypoventilation. Between 4% and 7.5% of patients with anti-NMDARe have both glial and neuronal surface antibodies. A study of 215 anti-NMDARe patients, in which only 22 patients were positive for MOG-Ab, concluded that double antibody positivity is rare (4). In addition, Martinez-Hernandez (5) reported that patients with anti-NMDARe combined with MOG-Ab positivity have additional clinical radiological characteristics that may affect prognosis.

Despite previous case reports on the coexistence of FLAMES with anti-NMDARe, the clinical features, ancillary tests, treatment, and prognosis of these combined conditions have not been reviewed in detail. This paper reports a case of FLAMES combined with anti-NMDARe. In addition, we systematically review cases reported in the literature to provide information on the clinical symptoms, magnetic resonance imaging (MRI) findings, electroencephalographic (EEG) abnormalities, and treatment and prognosis of this rare overlap syndrome.

## Materials and methods

**Figure 1:**
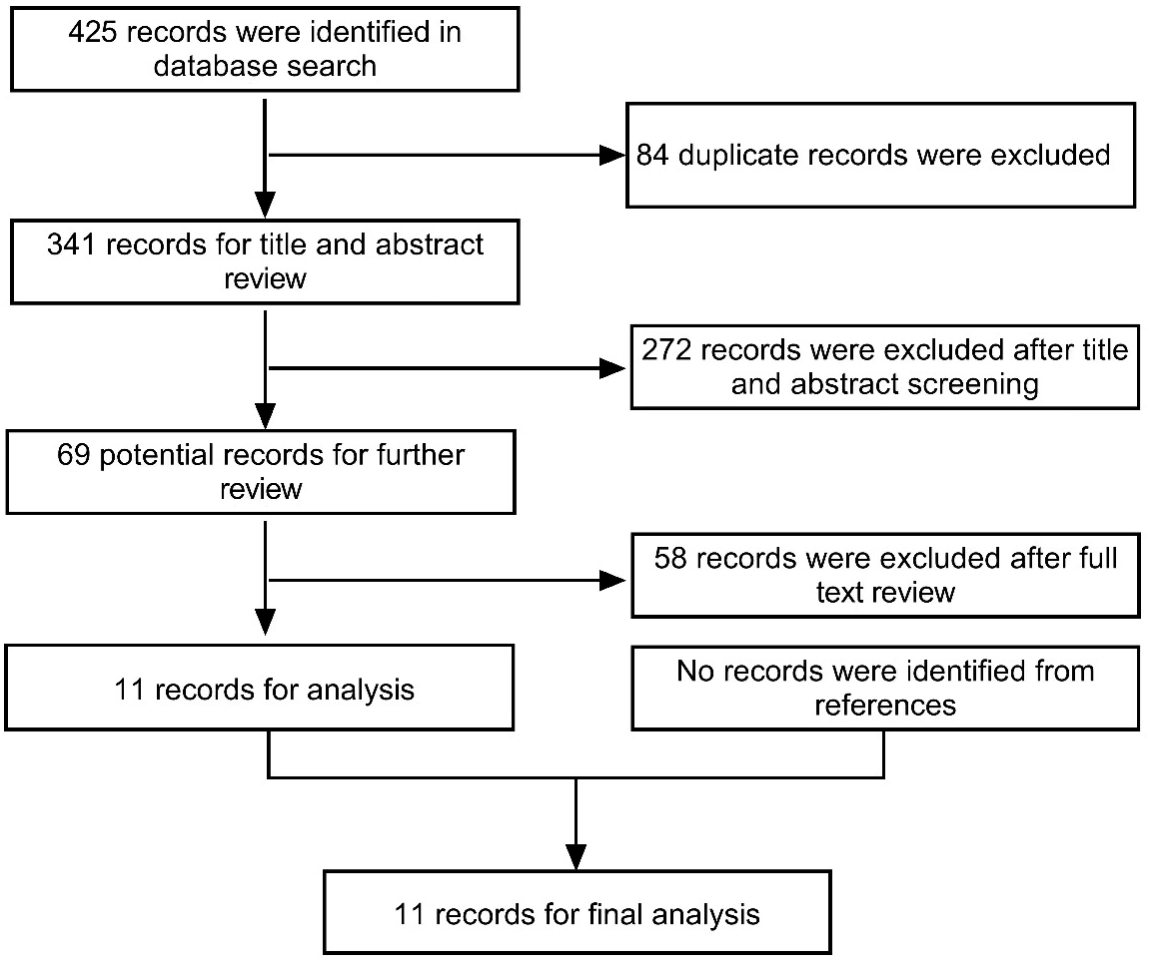
PRISMA flowchart.

In accordance with the Preferred Reporting Items for Systematic Reviews and Meta-Analyses (PRISMA) guidelines, we searched the PubMed database for publications up to September 15, 2022 that contained the terms “NMDAR” and “MOG”, or “NMDAR” and “Demyelination” and “MOG” and “Encephalitis”, in order to identify articles reporting on patients with coexisting FLAMES and anti-NMDARe (**Figure 1**). The inclusion criteria were as follows: (1) patients were positive for anti-NMDAR antibodies in the CSF and MOG antibodies in the serum; (2) the course of the disease included an episode of FLAMES; and (3) complete data were available on clinical symptoms, imaging, and treatment. Patients lacking a detailed clinical course description or radiological and laboratory information were excluded from the study. One new case diagnosed in our hospital was also included in the study. Information on the enrolled cases, including our case, is listed in **Table 1**. The study was approved by the Ethics Committee of the First Hospital of Jilin University. Written informed consent was obtained from the patients for the release of data and accompanying images.

**Table 1:** Summary of cases included in this study.

No.	Age	Sex	Clinical symptoms					CSF			MOG-ab		NMDAR		MRI		EEG	Relapse	Treatment
			Fever	Headache	Epilepsy	Visual impairment	Other symptoms	ICP	WC	Protein	CSF	Serum	CSF	Serum	Cortical FLAIR hyperintensity	Infratentorial lesion			
1	31	M	+	+	GTCS	+	Right hemiplegia, ataxia, ON		142	0.67		1:320	1:1	–	Right temporal, parietal, occipital	Midbrain, pons, medulla oblongata	Rt hemispheric-onset seizures	4	IVIG,IVMP,PSL/AZA,MMF
2	20	F	+	+	GTCS	+	Lower limb weakness, consciousness disorder, ON		29	0.38	1:16	1:128	+		Bilateral medial frontal lobe, cingulate	Pons	Diffuse slow wave (3HZ)	1	IVIG, IVMP, PE
3	36	F	+	+	Fas	–	Aphasia, disorientation, consciousness disorder, ataxia, central hypoventilation, involuntary movement during argument, delusion, depression		128	0.40		+	1:20		Bilateral medial frontal lobe	Cerebellum		1	IVIG, IVMP, PE
4	10	F	+	+	Fas	+	Involuntary movement during argument, consciousness disorder, ON	360	1	0.32		+	1:1	–	Right frontal, parietal		Spike and slow-wave discharges in the right hemisphere	4	IVIG,IVMP,PSL/RTM,MTX
5	21	M	+	+	GTCS	+	ON				–	1:32	1:32	+	Right frontotemporal lobe, meningeal enhancement		Normal	1	IVMP,IVIG,PSL
6	37	M	–	+	GTCS	–	Left limb weakness		11	0.48	1:10	1:10	1:1	1:1	Bilateral medial frontal lobe, meningeal enhancement		Normal	0	PSL
7	39	M	+	+	UEPC	–	Orientation, memory impairment, central hypopnea, delusions		112	0.44	+	1:1024	+		Bilateral medial frontal lobe, cingulate		Diffuse slow wave	2	IVMP,IVIG
8	38	M	+	+	GTCS	–	Restless	380	396	1.42	1:10	1:32	1:10	–	Left hemispheric		Left frontotemporal lobe apical–slow-wave activity	0	IVMP,IVIG,PSL/MMF
9	28	M	+	+	GTCS	+	ON	250	610	1.28	1:32	1:10	1:10		Left frontal		Scattered slow wave	0	IVMP,PSL/MMF
10	20	M	+	+	GTCS	+	ON	240	590	0.82		1:32	1:10	1:10	Right parietal				IVMP
11	38	M	–	–	GTCS	+	Left hemiplegia, aphasia, consciousness disorder	275	88	0.48	+	+	+	+	Bilateral cerebral cortex	Brain stem		2	IVMP,IVIG,PSL/MMF
12	30	M	+	+	GTCS	–	Consciousness disorder, aphasia, restless	150	268	0.37	1:100	1:1000	1:1+	–	Right frontal, meningeal enhancement		Left temporal apical wave, right temporal slow wave	0	IVMP,IVIG,LEV, PSL/CTX

M, male; F, female; ON, optic neuritis; ICP, intracranial pressure; FAS, focal aware motor onset seizure; GTCS, generalized tonic–clonic seizure; RTM, rituximab; LEV, levetiracetam; AZA, azathioprine; MMF, mycophenolate mofetil; CTX, cyclophosphamide; PSL, prednisolone; IVMP, intravenous methylprednisolone; IVIG, intravenous human immunoglobulin, UEPC, unexplained epilepsia partialis continua; D, days; Y, years; M, months. Case1 (6); case2 (7); case3 (8); case4 (9); case5 (10); case6 (11); case7 (12); case8 (13); case9 (14); case10 (15); case11 (16).

## Case report

**Figure 2:**
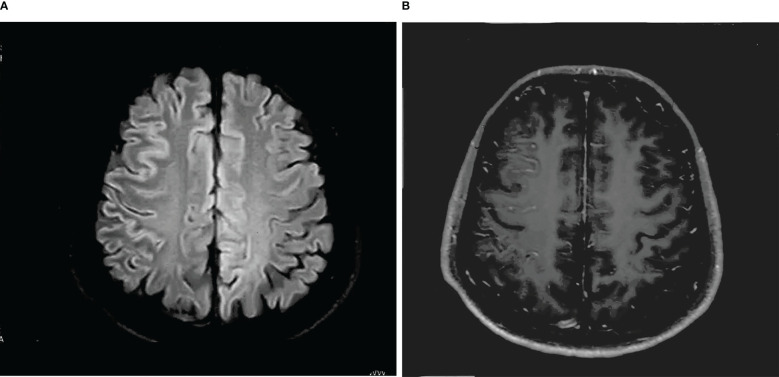
**(A)** T-2 FLAIR hyperintensity in the right frontal cortex, swelling of brain tissue, and shallowing of the cerebral sulcus. **(B)** Abnormal enhancement of the pia mater in the right frontal lobe.

**Figure 3:**
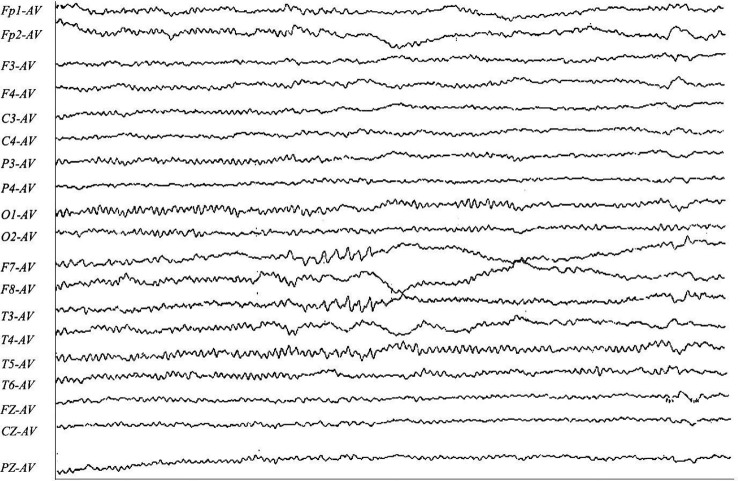
F7-AV, T3-AV, T5-AV: the left temporal region exhibits predominantly sharp-wave activity in the anterior and middle temporal regions, with some involvement of the posterior temporal region. F8-AV, T4-AV: The right anterior middle temporal region exhibits slow-wave activity.

A 30-year-old man initially complained of headaches and memory loss. After 15 days, the patient suffered a generalized tonic–clonic seizure with a five-minute period of loss of consciousness. Neurological examination on admission showed no significant symptoms except for verbal confusion, agitation, and abnormal mental behavior. MRI showed a FLAIR-hyperintense lesion and abnormal enhancement in the right frontal cortex (**Figure 2**), while EEG revealed slow-wave activity in the right temporal lobe, sharp-wave activity in the left temporal lobe, and a limited decrease in brain function (**Figure 3**). A CSF examination indicated 100×10^6^/L erythrocytes, 268×10^6^/L leukocytosis, 98% mononuclear cells, and a protein concentration of 0.37g/L. CSF gram staining, ink staining, and bacterial and fungal cultures were negative. The patient was presumed to have viral encephalitis and was treated empirically with ganciclovir and levetiracetam. Five days after admission, the patient developed fever and persistent agitation. Cellular immunoassay results were positive for MOG antibodies (serum, 1:1000; cerebrospinal fluid, 1:100), autoimmune encephalitis-associated autoantibodies, and anti-NMDAR antibodies (serum, 1:10; cerebrospinal fluid, 1:10). autoimmune encephalitis-associated autoantibodies, and anti-NMDAR antibodies (serum: cerebrospinal fluid, 1:10). The patient was then administered intravenous immunoglobulin (0.4g/kg/d for 5 days) and intravenous methylprednisolone (1g/d for 3d, 0.5g/d for 3d, and 0.12g/d for 3d). This resulted in gradual improvement in the above symptoms. Repeat cerebral MRI 28 days after admission showed the disappearance of the FLAIR-hyperintense lesion in the right frontal cortex, while a CSF examination indicated 20×10^6^/L leukocytes and 0.30 g/L protein. A fixed cell assay was positive for anti-NMDAR antibodies (CSF, 1:1+) and MOG antibodies (serum 1:10+, CSF 1:1+). In addition, results were negative for all of the following antibodies: anti-aquaporin-4 (AQP4), anti-NMDAR, anti-leucine-rich glioma inactivated 1 (LGI1), anti-contactin-associated protein-like 2 (CASPR2), α-amino-3-hydroxy-5-methyl-isoxazolepropionic acid receptor (AMPAR), and gamma-aminobutyric acid (GABA) receptor. After discharge, the patient was advised to take oral prednisolone and cyclophosphamide. We followed up with the patient after two years, and he had returned to work with no recurrence of symptoms.

## Results of the literature search

### Demographics and clinical characteristics

The search of the PubMed database identified a total of 425 studies, 11 of which were included in this study (n=12 patients with the addition of our case). Nine of the 12 patients were male (75%), with the age of onset ranging from 10 to 39 years (mean: 29 years).

The most frequent clinical manifestation of the overlay of FLAMES with anti-NMDARe was epilepsy (100%), followed by generalized tonic spastic seizures (75%), focal seizures (16.7%), and unknown forms of seizure (8.3%). Other common characteristics were headache (92%), fever (83%), decreased vision (58%), disorders of consciousness (42%), aphasia (33%), limb weakness (33%), psychiatric symptoms such as delirium, agitation, hallucinations, and babbling (33%), central hypoventilation (17%), cognitive impairment, disorientation, and ataxia (**Table 2**). Six patients also had ON. No patient had a teratoma or tumors.

**Table 2:** Demographic and clinical data on all patients.

Sex (male), n (%)	9 (75%)
Age (years), average	29
Clinical presentation, n (%)	
Epilepsy	12 (100%)
Headache	11 (92%)
Fever	10 (83%)
Vision loss	7 (58%)
Disorder of consciousness	5 (42%)
Aphasia	4 (33%)
Limb weakness	4 (33%)
Psychiatric symptoms	4 (33%)
Central hypoventilation	2 (17%)
CSF	
Pressure	262.5 mmH_2_O
WBC, median	128 × 10^6^ / L
Protein, median	0.48g/L
MOG, median	1:16
NMDAR, median	1:10
Serum	
MOG, median	1:32
FLAIR cortical hyperintensity	
Unilateral cortical	7 (58%)
Bilateral medial frontal lobes	4 (33%)
Infratentorial lesion	4 (33%)

### Laboratory characteristics

CSF examinations showed leukocytes in the range of 1–610×10^6^/L (median: 128×10^6^/L), with this increase caused predominantly by monocytes. Protein levels were normal or slightly elevated, with a median of 0.48 g/L. Six patients had intracranial pressure results indicating CSF pressure ranging from 150 to 380 mmH_2_O, with a median of 262.5 mmH_2_O (**Table 2**).

All patients were positive for CFS anti-NMDAR antibodies and serum anti-MOG antibodies. CSF anti-NMDAR antibody titers ranged between 1:1 and 1:32 (median: 1:10), while MOG antibody titers in serum ranged from 1:10 to 1:1024 (median: 1:32). Some patients also tested positive for MOG antibodies in CSF, with titers ranging from 1:10 to 1:100 (median: 1:16). In one patient, mGluR5 antibodies were concurrently detected in the cerebrospinal fluid and serum.

### EGG

EEG results were obtained for 9 patients, 4 of whom exhibited slow-wave activity; 2 exhibited pike-slow waves, 1 exhibited an epileptiform pattern, and 2 patients had normal EEG results. None of the patients exhibited the characteristic abnormal δ brush of NMDARe.

### Neuroimaging findings

All 12 patients had a FLAIR-hyperintense lesion with anti-MOG-associated encephalitis, with seizures occurring during the disease episode. Seven patients presented with unilateral cortical FLAIR-hyperintense lesions, usually involving the frontal, temporal, and parietal lobes, some with meningeal enhancement. Five of the 12 patients had lesions at other sites (e.g., the brainstem, corpus callosum, and frontal orbital gyrus). Five patients (41.7%) showed bilateral cortical FLAIR-hyperintense lesions, four of which were in the bilateral medial frontal lobes. One patient had a hyperintense brainstem lesion before FLAME, and in 4 patients this occurred after the development of the disease.

### Treatment and outcomes

First-line treatments included IVMP (11/12), IVIG (9/12), and plasma exchange (2/12). Eight patients received a combination of IVMP+IVIG. Of the 12 patients, six recovered with first-line treatment. The second line of treatment included mycophenolate mofetil (4/12), azathioprine (1/12), cyclophosphamide (1/12), rituximab (1/12), and methotrexate (1/12). Seven patients relapsed during the disease episode, with the median number of relapses being two. Follow-up data were collected for four patients over a mean time period of 18.5 months. Only one patient experienced residual visual impairment, although this did not affect their daily work. The remaining 11 patients had no significant sequelae.

## Discussion

FLAMES is a rare clinical phenotype of MOGAD that has been reported since 2017 (17). Fujimori et al. suggested that the specific role of MOG-ab in MOG cerebral cortical encephalitis (CCE) may have relevance to autoimmune encephalitis such as NMDARe, although its mechanism of action remains unclear (18). Vega’s study on MOG-positive non-ADEM encephalitis showed that 40% of patients had concomitant NMDAR-abs in the CSF (19). Currently, many scholars consider positivity for both antibodies to be relevant to oligodendrocytes. Both MOG receptors and NMDAR can be expressed on the surface of oligodendrocytes, which may explain the double antibody positivity (20). In addition, the viral infection causes the blood–brain barrier to be disrupted, triggering subsequent inflammatory and immune responses. Mariotto’s study (21) showed that 45% of patients with MOG antibody-related disease had prodromal symptoms or an infectious process. There is evidence that anti-NMDARe can be triggered by viral encephalitis, particularly the herpes simplex virus (22). Four patients in our study had flu-like symptoms before onset, and HHV-7 was detected in one patient using second-generation sequencing (14).

In this study, we have discussed the clinical features, neuroimaging features, and prognosis of 12 cases of coexistence of FLAMES and anti-NMDARe. Anti-NMDARe usually occurs in female patients, while the overlap syndrome is more common in male patients (75%). The common clinical manifestations of the overlap syndrome are epilepsy, headache, fever, vision impairment, psychiatric symptoms, aphasia, and (less frequently) central hypoventilation, involuntary chewing movements at the corners of the mouth, and loss of consciousness. ON is rare in anti-NMDARe patients but has been reported in overlap syndrome patients. Compared with classic anti-NMDARe, patients with the overlap syndrome have milder clinical symptoms (23). In our study, 10 patients initially had FLAMES while two patients had NMDARe. It is difficult to distinguish this overlap syndrome from FLAMES alone on the basis of clinical presentation. Previously, it had been concluded that anti-NMDARe in female patients was associated mostly with teratomas. However, no tumors were found in any of the patients in this study. Therefore, tumors are unlikely to be the primary trigger for NMDARe in FLAMES patients. Titulaer’s study (11) concluded that the double positivity of the anti-NMDAR antibody and MOG-ab represented an autoimmune rather than a tumor trigger.

Overlap syndrome patients have CSF findings similar to those observed in patients with FLAMES alone. It is possible to observe elevated intracranial pressure, elevated leukocytes and erythrocytes, and mildly elevated protein levels. CSF titers of the anti-NMDAR antibody in overlap syndrome patients were in the range 1:1–1:32, with a median of 1:10. Overlap syndrome patients had lower antibody titers than seen in anti-NMDARe alone, which could explain the mostly mild symptoms of anti-NMDARe in these patients. Gresa-Arribas et al. (24) concluded that anti-NMDAR antibody titers are positively associated with poor prognosis. All the patients in our study with anti-NMDARe had mild clinical symptoms and achieved a good prognosis at subsequent follow-up. Anti-NMDARe is one of the most common types of autoimmune encephalitis, with the associated EEG abnormalities mainly being generalized slow waves, followed by focal slow waves in the frontal or anterior temporal regions (25, 26). The EEG abnormalities in FLAMES patients are seen in the central, parietal, posterior temporal, or occipital regions. The frontal and parietal lobes are usually involved in epileptic activity, whereas the limbic system is not (26). EEG may be normal during the interictal period, and in patients with FLAMES, EEG may differ from that measured in other types of autoimmune encephalitis. In cases of focal epilepsy, FLAMES patients can exhibit slow waves or epileptic waves in the parietal and occipital lobes, which is rare in patients with other autoimmune encephalitides. These EEG characteristics can prompt clinicians to test for MOG antibodies. Tokumoto’s (26) EEG study on FLAMES patients concluded that the abnormal slow waves mainly arose from the posterior temporal region and that no abnormalities were recorded in the anterior temporal region. This characteristic is distinct from the results observed for the patients in our study, whose EEGs suggested predominantly sharp waves in the left temporal region with some involvement of the posterior temporal region, while the right side exhibited slow-wave activity in the anterior middle temporal region. FLAMES in which EEG shows sharp–slow waves in the anterior temporal region may indicate anti-NMDAR antibody positivity. However, no reports relevant to this were identified in the current study, and further studies are therefore necessary.

The current study analyzed a total of 12 cases of FLAMES combined with anti-NMDARe. Four of the 12 patients showed bilateral medial frontal FLAIR-hyperintense lesions. Fujimori suggested that the presence of anti-NMDARe and MOG-ab may result in the onset of bilateral medial frontal lobe CCE almost simultaneously, and this possibility was confirmed in these four patients. Cherian et al. (24) pointed out that contrast enhancement in the bilateral medial frontal lobe, especially the bilateral cingulate gyrus, can be an imaging feature for the coexistence of dual antibodies to MOG and NMDAR. The four aforementioned patients in our study developed subtentorial demyelinating lesions during the course of the disease. Consistent with the hypothesis of Ren et al. (16), in patients with recurrent CNS demyelination, especially MRI brainstem lesions and cortical involvement, double positivity for MOG-ab and NMDAR antibodies should be considered. Furthermore, Vega’s latest study showed that a significantly higher proportion of patients with FLAMES combined with non-ADEM encephalitis (13/25) presented with NMDAR antibodies in their CSF compared to the proportion among patients with FLAMES alone (2/13) (19). This further demonstrates that when FLAMES patients show infratentorial lesions on MRI, this represents an alert to check for the presence of the overlay syndrome. Such potentially unique imaging characteristics therefore provide clinicians with greater insight into the diagnosis of the overlap syndrome.

Vega’s study concluded that patients with the overlay syndrome have a higher rate of relapse than patients with FLAMES alone (19). Patients with the overlay syndrome usually have a positive response to steroids, with the clinical symptoms of most patients being relieved within a short time after receiving IVIG and IVMP treatment. However, if steroids are discontinued, or reduced too quickly, a relapse may occur. Patients can then receive repeat first-line therapy, while second-line immunotherapy such as rituximab, mycophenolate, and azathioprine should be added. In general, it has been reported that all patients have recovered after appropriate treatment, with no significant sequelae remaining (6, 27). In addition, immunosuppression therapy may reduce relapse in patients.

## Conclusion

The rate of coexistence of FLAMES and anti-NMDARe may be underestimated. It is difficult to distinguish patients with FLAMES alone from those with the overlap syndrome based on clinical characteristics. Patients diagnosed with FLAMES should be actively screened for anti-NMADR antibodies if MRI shows bilateral medical frontal lobe FLAIR-hyperintense lesions. Overlap syndrome patients should be treated with extended immunotherapy, which can reduce relapses and offer a good prognosis.

## Ethics statement

Written informed consent was obtained from the participant/patient(s) for the publication of this case report.

## Author contributions

JC and J-XY: designed the study. J-XY, M-MY, C-HG, and Y-JH: interpreted the data. J-XY: drafted the manuscript. All authors revised the manuscript critically for important intellectual content and gave final approval of the version to be published. All authors contributed to the article and approved the submitted version.

## References

[1] Banwell B Bennett JL Marignier R Kim HJ Brilot F Flanagan EP. Diagnosis of myelin oligodendrocyte glycoprotein antibody-associated disease: International MOGAD panel proposed criteria. Lancet Neurol (2023) 22(3):268–82. doi: 10.1016/S1474-4422(22)00431-8 36706773

[2] Budhram A Mirian A Sharma M. Meningo-cortical manifestations of myelin oligodendrocyte glycoprotein antibody-associated disease: Review of a novel clinico-radiographic spectrum. Front Neurol (2022) 13:1044642. doi: 10.3389/fneur.2022.1044642 36341089 PMC9630470

[3] Reijerkerk A Kooij G van der Pol SM Leyen T Lakeman K van Het Hof B. The NR1 subunit of NMDA receptor regulates monocyte transmigration through the brain endothelial cell barrier. J Neurochem (2010) 113(2):447–53. doi: 10.1111/j.1471-4159.2010.06598.x 20085611

[4] Hamid SHM Whittam D Saviour M Alorainy A Mutch K Linaker S. Seizures and encephalitis in myelin oligodendrocyte glycoprotein IgG disease vs aquaporin 4 IgG disease. JAMA Neurol (2018) 75(1):65–71. doi: 10.1001/jamaneurol.2017.3196 29131884 PMC5833490

[5] Martinez-Hernandez E Guasp M García-Serra A Maudes E Ariño H Sepulveda M. Clinical significance of anti-NMDAR concurrent with glial or neuronal surface antibodies. Neurology (2020) 94(22):e2302–10. doi: 10.1212/WNL.0000000000009239 32161029

[6] Zhou L ZhangBao J Li H Li X Huang Y Wang M. Cerebral cortical encephalitis followed by recurrent CNS demyelination in a patient with concomitant anti-MOG and anti-NMDA receptor antibodies. Mult Scler Relat Disord (2017) 18:90–2. doi: 10.1016/j.msard.2017.09.023 29141829

[7] Nagata S Nishimura Y Mitsuo K. [A case of anti-myelin oligodendrocyte glycoprotein (MOG) and anti-N-methyl-D-aspartate (NMDA) receptor antibody-positive encephalitis with optic neuritis]. Rinsho Shinkeigaku (2018) 58:636–41. doi: 10.5692/clinicalneurol.cn-001194 30270341

[8] Aoe S Kokudo Y Takata T Kobara H Yamamoto M Touge T. Repeated anti-N-methyl-D-aspartate receptor encephalitis coexisting with anti-myelin oligodendrocyte glycoprotein antibody-associated diseases: A case report. Mult Scler Relat Disord (2019) 35:182–4. doi: 10.1016/j.msard.2019.08.002 31398656

[9] Taraschenko O Zabad R. Overlapping demyelinating syndrome and anti-N-methyl-D-aspartate receptor encephalitis with seizures. Epilepsy Behav Rep (2019) 12:100338. doi: 10.1016/j.ebr.2019.100338 31737864 PMC6849071

[10] Du L Wang H Zhou H Chang H Wei Y Cong H. Anti-NMDA receptor encephalitis concomitant with myelin oligodendrocyte glycoprotein antibody diseases: A retrospective observational study. Med (Baltimore) (2020) 99(31):e21238. doi: 10.1097/MD.0000000000021238 PMC7402765 32756102

[11] Titulaer MJ Höftberger R Iizuka T Leypoldt F McCracken L Cellucci T. Overlapping demyelinating syndromes and anti–N-Methyl-D-Aspartate receptor encephalitis. Ann Neurol (2014) 75(3):411–28. doi: 10.1002/ana.24117 PMC4016175 24700511

[12] Fujimori J Takahashi T Kaneko K Atobe Y Nakashima I. Anti-NMDAR encephalitis may develop concurrently with anti-MOG antibody-associated bilateral medial frontal cerebral cortical encephalitis and relapse with elevated CSF IL-6 and CXCL13. Mult Scler Relat Disord (2021) 47:102611. doi: 10.1016/j.msard.2020.102611 33160141

[13] Fu J Peng L Yang Y Xie Y Li Z Rong B. Case report: Overlapping syndrome mimicking infectious meningoencephalitis in a patient with coexistent MOG, NMDAR, mGluR5 antibody positivity. Front Immunol (2022) 13:919125. doi: 10.3389/fimmu.2022.919125 35990698 PMC9389075

[14] Li S Wang M Li H Wang J Zhang Q Zhou D. Case report: Overlapping syndrome of anti-NMDAR encephalitis and MOG inflammatory demyelinating disease in a patient with human herpesviruses 7 infection. Front Immunol (2022) 13:799454. doi: 10.3389/fimmu.2022.799454 35529871 PMC9074690

[15] Yao T Zeng Q Xie Y Bi F Zhang L Xiao B. Clinical analysis of adult MOG antibody-associated cortical encephalitis. Mult Scler Relat Disord (2022) 60:103727. doi: 10.1016/j.msard.2022.103727 35320766

[16] Ren BY Guo Y Han J Wang Q Li ZW. Case report: Anti-NMDAR encephalitis with anti-MOG CNS demyelination after recurrent CNS demyelination. Front Neurol (2021) 12. doi: 10.3389/fneur.2021.639265 PMC7943444 33716942

[17] Ogawa R Nakashima I Takahashi T Kaneko K Akaishi T Takai Y. MOG antibody-positive, benign, unilateral, cerebral cortical encephalitis with epilepsy. Neurol: Neuroimmunol Neuroinflamm (2017) 4(2):e322. doi: 10.1212/NXI.0000000000000322 28105459 PMC5241006

[18] Fujimori J Takai Y Nakashima I Sato DK Takahashi T Kaneko K. Bilateral frontal cortex encephalitis and paraparesis in a patient with anti-MOG antibodies. J Neurol Neurosurg Psychiatry (2017) 88(6):534–6. doi: 10.1136/jnnp-2016-315094 28209651

[19] Vega E Arrambide G Olive G Castillo M Felipe-Rucián A Tintoré M. Non-ADEM encephalitis in patients with myelin oligodendrocyte glycoprotein antibodies: a systematic review. Eur J Neurol (2023). doi: 10.1111/ene.15684 36704861

[20] Karadottir R Cavelier P Bergersen LH Attwell D. NMDA receptors are expressed in oligodendrocytes and activated in ischaemia. Nature (2005) 438:1162–6. 10.1038/nature04302 PMC1416283 16372011

[21] Mariotto S Ferrari S Monaco S Benedetti MD Schanda K Alberti D. Clinical spectrum and IgG subclass analysis of anti-myelin oligodendrocyte glycoprotein antibody-associated syndromes: a multicenter study. J Neurol (2017) 264(12):2420–30. doi: 10.1007/s00415-017-8635-4 PMC5688213 29063242

[22] Pruss H. Postviral autoimmune encephalitis: manifestations in children and adults. Curr Opin Neurol (2017) 30:327–33. doi: 10.1097/WCO.0000000000000445 28234798

[23] Fan S Xu Y Ren H Guan H Feng F Gao X. Comparison of myelin oligodendrocyte glycoprotein (MOG)-antibody disease and AQP4-IgG-positive neuromyelitis optica spectrum disorder (NMOSD) when they co-exist with anti-NMDA (N-methyl-D-aspartate) receptor encephalitis. Mult Scler Relat Disord (2018) 20:144–52. doi: 10.1016/j.msard.2018.01.007 29414288

[24] Cherian A Divya KP Shetty SC Kannoth S Thomas B. Coexistent MOG, NMDAR, CASPR2 antibody positivity: Triumph over the triumvirate. Mult Scler Relat Disord (2020) 46:102468. doi: 10.1016/j.msard.2020.102468 32906000

[25] Dalmau J Gleichman AJ Hughes EG Rossi JE Peng X Lai M. Anti-NMDA-receptor encephalitis: case series and analysis of the effects of antibodies. Lancet Neurol (2008) 7(12):1091–8. doi: 10.1016/S1474-4422(08)70224-2 PMC2607118 18851928

[26] Tokumoto K Nishida T Kawaguchi N Kaneko K Takahashi T Takahashi Y. Electroclinical features of seizures in myelin oligodendrocyte glycoprotein antibody-associated cerebral cortical encephalitis: A case report and literature review. Seizure (2022) 98:13–8. doi: 10.1016/j.seizure.2022.04.001 35397246

[27] Zhou J Tan W Tan SE Hu J Chen Z Wang K. An unusual case of anti-MOG CNS demyelination with concomitant mild anti-NMDAR encephalitis. J Neuroimmunol (2018) 320:107–10. doi: 10.1016/j.jneuroim.2018.03.019 29661538

